# The effects of brain endurance training on physical endurance performance under mental fatigue and non-fatigued conditions: a systematic review and meta-analysis

**DOI:** 10.3389/fpsyg.2026.1858200

**Published:** 2026-09-11

**Authors:** Ning Wang, Zhengxiang Chen, Kewei Zhao

**Affiliations:** China Institute of Sport Science, Beijing, China

**Keywords:** brain endurance training, endurance performance, mental fatigue, meta-analysis, systematic review

## Abstract

**Background:**

Mental fatigue impairs physical endurance performance. Brain Endurance Training (BET), which combines cognitive tasks with physical training, may help alleviate these negative effects.

**Purpose:**

To systematically review and meta-analyze the effects of BET on physical endurance performance under mental fatigue and control (non-fatigued) conditions in athletes and healthy adults.

**Methods:**

This PRISMA 2020-compliant review was registered in PROSPERO (CRD420251036649). PubMed, Web of Science Core Collection, Scopus, and the Cochrane Library were searched from inception through 5 August 2026. Eligible randomized controlled trials compared BET plus physical training with physical training alone in athletes or healthy adults and reported aerobic, anaerobic, or muscular endurance outcomes. Risk of bias was assessed for specific endurance outcomes using RoB 2. Random-effects meta-analyses used standardized mean differences (SMDs) with 95% confidence intervals (CIs), with all effects oriented so that positive values favored BET. Heterogeneity was quantified using *I*^2^, leave-one-out analyses examined influential experiments, and certainty was assessed using GRADE. Funnel plots and Egger's regression were used only as exploratory assessments of small-study effects because each synthesis contained fewer than 10 independent experiments.

**Results:**

Ten RCT articles comprising 12 independent experiments were included. Under mental-fatigue conditions, eight independent experiments (*n* = 275) showed a positive overall effect of BET on endurance performance (SMD = 0.82, 95% CI 0.33 to 1.30; *p* = 0.001; *I*^2^ = 69%). Under non-fatigued conditions, nine independent experiments (*n* = 315) yielded an imprecise and highly heterogeneous estimate (SMD = 0.73, 95% CI −0.03 to 1.49; *p* = 0.058; *I*^2^ = 89%). A formal moderator test did not identify a statistically significant difference between test states (contrast = 0.59, 95% CI −0.30 to 1.48; *p* = 0.120). Overall RoB 2 judgments raised some concerns, and GRADE certainty ranged from very low to moderate.

**Conclusions:**

BET may improve endurance performance after mental-fatigue induction, although the magnitude remains uncertain. Evidence under non-fatigued conditions is inconclusive, and the formal moderator test did not confirm a difference between test states. Larger independent trials with prespecified outcomes and complete reporting are needed.

**Systematic review registration:**

https://www.crd.york.ac.uk/PROSPERO/view/CRD420251036649, identifier: CRD420251036649.

## 1 Introduction

Fatigue can be broadly classified as physical or mental. Mental fatigue is a psychobiological state caused by prolonged, demanding cognitive activity and is characterized by increased subjective tiredness, reduced motivation and attention, heightened perceived effort, and impaired cognitive performance (Dallaway et al., 2022b). Neuroimaging studies have found alterations in brain activity patterns, such as decreased prefrontal cortex efficiency, which underlie these symptoms (Ishii et al., 2014).

Previously, mental fatigue was thought to impair cognitive performance, but as research has progressed, it has become a consensus among a growing number of researchers that mental fatigue leads to reduced motor performance (Van Cutsem et al., 2017). Emerging evidence from neuroscience suggests that mental fatigue triggers compensatory brain mechanisms, such as weakened connectivity between the frontal and parietal lobes, which results in diminished cognitive control and subsequent motor deficits.

To further explore the connection between mental fatigue and athletic performance, researchers in recent years have employed cognitive task interventions to induce mental fatigue (Hassan et al., 2023; Lorcery et al., 2024; Goodman et al., 2025; Dallaway et al., 2022b; Wu et al., 2025). The sustained cognitive demands imposed by these tasks may alter the perceived cost of effort, reduce the willingness to remain engaged, and affect prefrontal cognitive-control processes.

Prior studies have demonstrated that mental fatigue adversely affects endurance performance (Martin et al., 2018), e.g., mental fatigue leads to a reduction in subjects‘ power in the Wingate test (MacMahon et al., 2014), and reduces cyclists' maximum cycling time (Marcora et al., 2009), and when performing a 5 km treadmill running, average speed also slows down (Pageaux et al., 2014). Additional indicators of endurance performance similarly demonstrate adverse effects from mental fatigue interventions. For instance, mental fatigue has been shown to impair anaerobic power and endurance jump performance, with alterations in kinetics and kinematics during jumps (Gonzalez et al., 2024). It also negatively affects sport-related motor skills and decision-making, extending beyond endurance to agility and precision tasks (Pageaux and Lepers, 2018; Holgado et al., 2020). Systematic reviews confirm that mental fatigue consistently impairs physical performance across various sports, with physiological explanations involving altered drive and motivation (Martin et al., 2018; Schiphof-Godart et al., 2018). Interestingly, endurance athletes appear more resistant to these effects compared to non-athletes, suggesting training adaptations that mitigate impairments (Daneshgar-Pironneau et al., 2025). Combined mental and physical fatigue exacerbates these issues, particularly in attention and reaction times during exercise (Mortimer et al., 2024). Interindividual variability exists, with some athletes showing greater susceptibility, though no clear predictors have been identified yet (Habay et al., 2023). Recent work emphasizes that mental fatigue does not always exacerbate central fatigue but can increase injury risk in sports (Schampheleer and Roelands, 2024).

To sustain endurance performance under mental fatigue or mitigate mental fatigue during exercise, several interventions to counteract mental fatigue in specific contexts are currently being evaluated (Proost et al., 2022), e.g., Franco-Alvarenga et al. used caffeine intake to allow subjects to complete a 20-km ride with a reduced time was shortened (Franco-Alvarenga et al., 2019), Fortes et al. used transcranial Direct Current Stimulation (tDCS) to counteract the decrease in swimming endurance due to mental fatigue (Fortes et al., 2022), In addition to nutritional supplements such as caffeine and electrical stimulation, mindfulness training (Cao et al., 2022) have also been found to reduce mental fatigue (McIntire et al., 2017; Sá Filho et al., 2025). Other promising countermeasures include nature exposure, mindfulness meditation, and binaural beats, which help restore cognitive resources (Sun et al., 2024). Brief mindfulness interventions have shown efficacy in reducing mental fatigue and improving tactical performance in sports like basketball (Cao et al., 2024; Dallaway et al., 2022a). Physiological coping includes nutritional and odor interventions to manage negative effects (Wu et al., 2024).

Brain Endurance Training (BET) combines cognitively demanding tasks with physical training, delivered sequentially, concurrently, or in alternating blocks. BET is intended to improve tolerance of combined cognitive and physical demands, and randomized trials have examined endurance performance both after mental-fatigue induction and in non-fatigued states (Staiano et al., 2023; Marcora et al., 2015). Recently, numerous original studies have explored how BET impacts athletic capabilities, particularly under mental fatigue conditions.

Two systematic reviews published in 2026 summarized the rapidly expanding BET literature (Qing et al., 2026; Yu et al., 2026). Yu et al. quantitatively compared outcomes assessed under fatigued and non-fatigued conditions, whereas Qing et al. reviewed physical and cognitive performance across athletes and physically active individuals. However, uncertainty remains regarding endurance-specific effect estimates, the handling of multi-experiment articles and correlated outcomes, and the extent to which mental-fatigue state modifies the BET effect. Accordingly, this review updates the literature search and separately evaluates the effects of BET on endurance performance under mental-fatigue and non-fatigued conditions, thereby extending the existing evidence.

## 2 Research methods

This systematic review was reported in accordance with the PRISMA 2020 statement (Page et al., 2021) and was registered in PROSPERO (CRD420251036649).

### 2.1 Information sources and search strategies

PubMed, Web of Science Core Collection, Scopus, and the Cochrane Library were searched from database inception through 5 August 2026. Reference lists of included articles and relevant reviews were also examined. Complete database-specific search strategies, fields, limits, exact search dates, and retrieval and export counts are provided in Supplementary File S1.

The strategy was developed from terminology used in known BET reports and expanded to include spelling and phrase variants for brain endurance, combined or concurrent cognitive-physical training, dual-task training, cognitive-motor training, and multimodal training, together with endurance and exercise-performance outcomes. Mental-fatigue terms were not mandatory because eligible trials could assess endurance under either mental-fatigue or non-fatigued conditions. No RCT filter was applied in PubMed, Web of Science, or Scopus; the Cochrane Library search was limited to Trials. Where supported, searches were limited to English.

All retrieved records were imported into Zotero Desktop 9.0.4. Duplicate records were identified using DOI, PMID, normalized title, and publication year, followed by manual review of highly similar titles.

### 2.2 Selection process

Two reviewers (NW and CZX) independently screened titles and abstracts and then assessed potentially eligible full texts. Disagreements were resolved by discussion and, when necessary, adjudication by a third reviewer (KWZ). Reasons for exclusion were recorded at the full-text stage for each report. The article-level exclusion log is provided in Supplementary File S3. Multiple reports from the same study and multiple independent experiments within one article were linked before data extraction.

### 2.3 Inclusion criteria

Studies were screened for eligibility using the PICOS method, which involved first reviewing the title and abstract of each article, followed by a full-text assessment, with selection conducted in accordance with the PICOS framework (Participants, Interventions, Comparisons, Outcomes, Study Design) as outlined in Table 1.

**Table 1:** Eligibility criteria based on the PICOS framework.

Form	Inclusion criteria	Exclusion criteria
P (Participants)	Athletes or healthy adults with no gender or training/competitive level restrictions	Injury, comorbidity or illness
I (Interventions)	Brain Endurance Training (BET) intervention ≥3 weeks, ≥1 session per week	Training methods other than cognitive tasks (e.g., purely physical or mental training)
C (Comparators)	Control group with physical training only	Absence of a control group
O (Outcomes)	Endurance performance (aerobic, anaerobic or muscular endurance) measured before and after the intervention	Unavailability of baseline and/or follow-up data
S (Study design)	Randomized controlled studies	Cross-sectional, observational or case studies

### 2.4 Data collection process

For each included article, two reviewers recorded the article identifier, independent experiment identifier, outcome and condition, group-level analyzed sample sizes, means, standard deviations, outcome direction, data source, and whether the same sample contributed to another effect estimate. Data extracted from figures were digitized using WebPlotDigitizer version 4.5 (Drevon et al., 2017). and independently checked. Disagreements were resolved by consensus. The complete effect-level dataset and the rationale for each included or excluded estimate are provided in Supplementary File S2.

#### 2.4.1 Brain endurance training interventions

BET methods (e.g., cognitive task type, training sequence), intervention duration (weeks, frequency), and training intensity were recorded. BET was defined as an intervention combining cognitive tasks with physical training for ≥3 weeks, ≥1 time per week. Intervention duration needed to be long enough to produce neural and muscular adaptations (Marcora et al., 2015). Based on the actual conditions of the included studies, we have categorized the organizational forms of the protocols into three types: sequential, where cognitive training and physical training are conducted consecutively in separate time periods, either with cognitive training followed by physical training or vice versa; concurrent or simultaneous, where cognitive tasks and physical training are performed synchronously within the same time period; and alternating, where cognitive task modules and physical training modules alternate within the same training session.

#### 2.4.2 Outcome measurements

Endurance outcomes were classified as aerobic, anaerobic, or muscular endurance. Outcomes were also classified by test state: mental-fatigue conditions, in which a cognitive task was used to induce mental fatigue before the endurance test, and non-fatigued conditions, in which no mental-fatigue induction preceded testing. The comparator in all analyses was the physical-training control group.

### 2.5 Risk of bias and exploratory assessment of small-study effects

Risk of bias was assessed for the specific endurance result contributing to each synthesis using the official Cochrane Risk of Bias 2 (RoB 2) tool. Potential small-study effects were explored using funnel plots and Egger's regression test. A two-sided *p* value < 0.05 was treated as a statistical signal of a small-study effect, not as proof of publication bias. Because each condition-specific synthesis contained fewer than 10 independent experiments, these analyses were considered exploratory and were not interpreted as definitive evidence for or against publication bias.

### 2.6 Statistical analyses

For parallel-group comparisons, treatment effects were expressed as Hedges' *g* (the standardized mean difference with a small-sample correction, reported as SMD) with 95% confidence intervals (CIs) (Higgins, 2023; Hedges, 1981), because endurance outcomes were measured on different scales. The primary calculation used the final measurement reported for each group: post-intervention values for outcomes assessed in the non-fatigued state and the corresponding final post-intervention values measured after the mental-fatigue induction for mental-fatigue outcomes. Change-from-baseline estimates were not combined with these final-value estimates in the primary models. Outcome directions were harmonized before analysis so that positive Hedges' *g* values consistently favored BET; outcomes for which lower values indicated better performance were multiplied by −1. Forest plots were labeled “Favors control” on the left and “Favors BET” on the right. Articles, independent experiments, participant samples, and effect estimates were treated as distinct units. Experiments reported within the same article were treated as independent only when they involved non-overlapping participants. Within each condition-specific synthesis, no participant sample contributed more than one effect estimate: when several eligible endurance outcomes shared participants, one estimate was retained and the selected outcome, data source, shared-sample status, and reason for selection were documented in Supplementary File S2. Alternative outcomes from the same participants were not entered as independent observations in the same model (Hedges et al., 2010). Mental-fatigue and non-fatigued estimates from the same participant sample were synthesized separately and their sample sizes were not added. Their dependence in the between-condition moderator analysis was addressed by clustering at the independent-experiment level (Tipton, 2015; Pustejovsky and Tipton, 2018). DerSimonian-Laird random-effects models were used for the primary condition-specific and endurance-domain syntheses; thus, between-experiment variance (τ^2^) was estimated using the DerSimonian-Laird method (DerSimonian and Laird, 1986). Statistical heterogeneity was evaluated using τ^2^, Cochran's *Q*, and *I*^2^; τ^2^ and *Q* were reported for the primary condition-specific syntheses, and *I*^2^ was reported throughout (Higgins et al., 2003). Prespecified subgroup analyses were conducted by endurance domain when at least two independent experiments were available. Leave-one-out analyses omitted each independent experiment in turn, and fixed-effect models were fitted as sensitivity analyses (Viechtbauer and Cheung, 2010).

Primary forest plots were generated in Review Manager (RevMan, version 5.4). Leave-one-out analyses and Egger's regression were conducted in R version 4.5.2 using metafor version 4.8-0 (R Core Team, 2025). To examine whether test state modified the effect of BET, a multilevel meta-regression was fitted with test state (mental-fatigue vs. non-fatigued conditions) as the moderator and a random intercept for independent experiment. All eligible condition-specific effect estimates were included in the moderator analysis. When the same independent experiment contributed estimates under both mental-fatigue and non-fatigued conditions, their statistical dependence was accounted for by clustering at the independent-experiment level (Hedges et al., 2010). The model was fitted using restricted maximum likelihood. Cluster-robust variance estimation was implemented with club Sandwich version 0.7.0 using the CR2 small-sample correction and Satterthwaite degrees of freedom at the independent-experiment level (Tipton, 2015; Pustejovsky and Tipton, 2018).

### 2.7 Certainty of evidence

Certainty of evidence was evaluated for each endurance outcome using GRADE (Schünemann et al., 2024). Randomized evidence started at high certainty and was downgraded, when appropriate, for risk of bias, inconsistency, indirectness, imprecision, and publication bias. Each downgrade or decision not to downgrade was justified in the Summary of Findings table. Because funnel-plot and Egger analyses involved fewer than 10 independent experiments, publication bias was rated as not reliably assessable.

### 2.8 Sensitivity analysis

Robustness was examined by omitting each independent experiment in turn and recalculating the pooled Hedges' *g* (reported as SMD), 95% CI, *p* value, τ^2^, and *I*^2^. Fixed-effect estimates were compared with the primary random-effects estimates. Influential changes were traced to the selected outcome, analyzed sample size, mean difference, and pooled SD recorded in Supplementary File S2, while estimates meeting the prespecified eligibility criteria were retained in the primary analysis.

## 3 Results

### 3.1 Study selection

**Figure 1:**
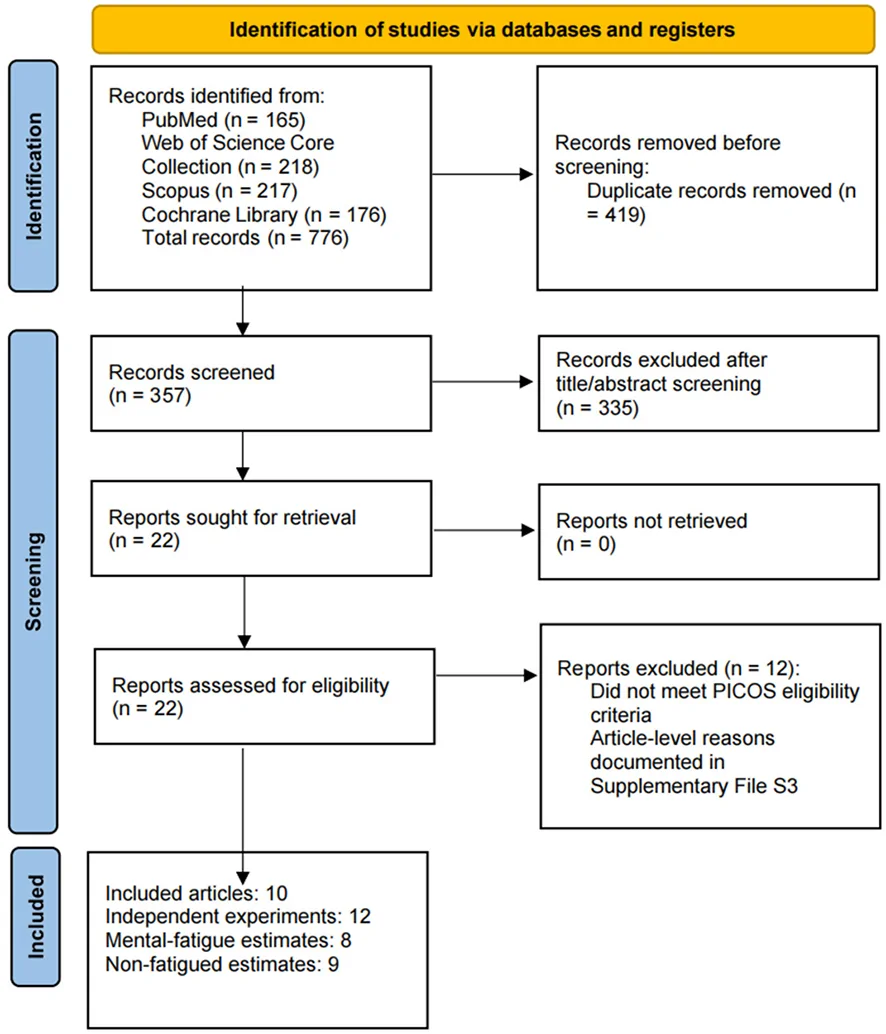
PRISMA flow diagram.

The database searches identified 776 records: 165 from PubMed, 218 from Web of Science, 217 from Scopus, and 176 from the Cochrane Library. After removal of 419 duplicate records, 357 unique records underwent title and abstract screening, of which 335 were excluded. Full texts were sought for 22 reports; all were retrieved and assessed for eligibility. Twelve full-text reports were excluded, and 10 articles comprising 12 independent experiments were included in the review and meta-analysis (Figure 1). Article-level reasons for full-text exclusion are provided in Supplementary File S3.

### 3.2 Characterization of the study

Participant and intervention characteristics are summarized in Tables 2A, B. Across 10 articles (12 independent experiments), the mental-fatigue and non-fatigued syntheses included eight estimates (*n* = 275) and nine estimates (*n* = 315), respectively. These totals were not combined because some participant samples contributed to both conditions.

**Table 2A:** Study and participant characteristics.

References	Sample size	Individual age	Gender	Group identity	Cognitive task	Number of training sessions
De Lima-Junior et al. (2023)	45	23.4 ± 2.9 years	Male	Endurance-trained men	Stroop mission	12 weeks, 3 times per week
Dallaway et al. (2024)	Exp1 29	23 ± 5 years	12 women, 17 men	Healthy Undergraduates	Stroop mission, 2-back mission	4 weeks, 3 times per week
	Exp2 29	21 ± 2 years	18 women, 11 men	Healthy Undergraduates	Switched stop visual, 2-back task multi-source interference task, time-load dual-bac task.	6 weeks, 3 times per week
Díaz-García et al. (2024)	91	29.42 ± 10.06 years	49 men, 42 women	Athletes with resistance training experience	Stroop mission	6 weeks, 5 times per week
Dallaway et al. (2021)	36	20 ± 2 years	15 women, 21 men	Healthy undergraduates	2-back mission, stroop mission	6 weeks, 4 times per week
Díaz-García et al. (2025)	24	65-78 years	Females	Sedentary older persons	The stroop mission.	3 times per week for 8 weeks
Dallaway et al. (2023)	24	20 ± 2 years	13 women, 11 men	Healthy Undergraduates	2-back mission, stroop mission	20 training sessions in 5 weeks
Staiano et al. (2022)	22	22.4 ± 4.3 years	Male	professional footballer	Choose any one of the flanker tasks, go-nogo tasks, or continuous performance tasks.	4–5 times per week for a total of 4 weeks
Staiano et al. (2023)	Exp1 26	29 ± 5 years old	Male	road cyclist	Flanker task, go-nogo task, continuous performance task	5 times a week for 6 weeks
	Exp2 24	25± 4 years old	Male	road cyclist	Flanker task, go-nogo task, continuous performance task	6 weeks, 3 times per week
Liu et al. (2026)	70	22.3 ± 4.2 years	Male	Sub-elite provincial-level competitive soccer players	Color-word stroop task; adaptive dual n-back task	6 weeks; 4 times a week
Rautu et al. (2026)	22	21.14 ± 1.25 years	14 men, 8 women	Recreational athletes	4 weeks; 3 week; 12 sessions	4 weeks; 3 times a week;

The 10 included articles comprised 12 independent experiments. The mental-fatigue and non-fatigued syntheses included outcome-specific analyzed samples of *n* = 275 and *n* = 315, respectively. These totals were not combined because some participant samples contributed outcomes under both test states. Sample sizes reported in Table 2 describe the samples reported by the original articles, whereas the meta-analyses included only participants who provided analyzable data for the selected endurance outcome in an eligible BET-control comparison.

**Table 2B:** Brain endurance training intervention characteristics.

References	BET organization	Physical training/comparator	Intensity/progression	Physical performance tests	Session composition/duration
De Lima-Junior et al. (2023)	Concurrent	Running training (60 per cent Vmax)	60 per cent of maximum aerobic speed	Exhaustion time (at 80% intensity of maximal aerobic rate)	Upgraded from 20 to 40 min depending on the week
Dallaway et al. (2024)	Exp1 Alternating	Two cycles of planks, squats, push-ups and static squats against the wall	70% of pre-test performance intensity increased by 5% per week deep squat jumps increased by 20% per week	Push-ups, wall sits, planks, squat jumps	24-min cognitive task and 24-min physical task
Dallaway et al. (2024)	Exp2 Alternating	Bobi jumps, planks, deep squat jumps, leg lifts and push-ups	Gradual increase (80–95 per cent maximum performance)	Completion of 20 min (four 5-min segments) of unassisted aerobics and mountain running.	15 min cognitive tasks, 15 min physical tasks
Díaz-García et al. (2024)	Sequential	Chest press, squat jumps	Four sets of chest press: 40% 6RM, four sets of deep squat jumps to exhaustion	Perform chest presses and deep squat jumps	27 min per session of cognitive tasks + resistance training
Dallaway et al. (2021)	Concurrent	Rhythmic Grip Training	Approximately 30% of the maximum voluntary contraction force (MVC)	Maximum voluntary contraction force (MVC)	12,000 units increased by 500 units per week
Díaz-García et al. (2025)	Sequential	Resistance Training+ Gait Training	Medium strength, 7–8 RPE	Gait test (6-min walk test), sit-to-stand test, arm curl test	20 min cognitive tasks + 25 min gait training + 20 min resistance training
Dallaway et al. (2023)	Sequential	30% Maximum grip strength training	RPE4–6	Maximum grip force per second for dynamic rhythmic handgrip tasks	Physical tasks are set at an initial target of 12,000 points and increase by 1,000 points per week.
Staiano et al. (2022)	Sequential	Standard Rugby Bodywork	Uniform distribution of heart rate intervals for each intensity	30–15 Intermittent Fitness Test (IFT) Trial Football Specific Reactive Agility Test (SRAG) Randomized Repetitive Sprint Ability Test (RSA)	20–30 min per session (cognitive training)
Staiano et al. (2023)	Exp1 Sequential	Cycling training + fitness training	Dominated by Zone2 (69–83% HR, min) interval	Time to Exhaustion (TTE) Test	Cognitive tasks were gradually increased from 30 to 60 min over the week
	Exp2 Sequential	Cycling training + fitness training	Dominated by Zone2 (69–83% HR, min) interval	Riding time trials, incremental simulation tests	Cognitive tasks are 30 min
Liu et al. (2026)	Sequential	Regular soccer training	Adaptive Stroop and dual n-back (70%−85% accuracy); physical intensity not reported	LSPT; LSST; reactive agility; repeated sprint; 30–15 IFT; HR; lactate	6 weeks; 24 × 30 min
Rautu et al. (2026)	Remote	Push, pull, and core training	3 × 6–8 reps; self-adjusted load; progressive overload; 2-min rest	Bench press and preacher curl at 75% estimated 1RM; squat jumps; RPE	4 weeks; 12 sessions; 33-min cognition + ~60-min exercise

### 3.3 Risk of bias and exploratory assessment of small-study effects

**Figure 2:**
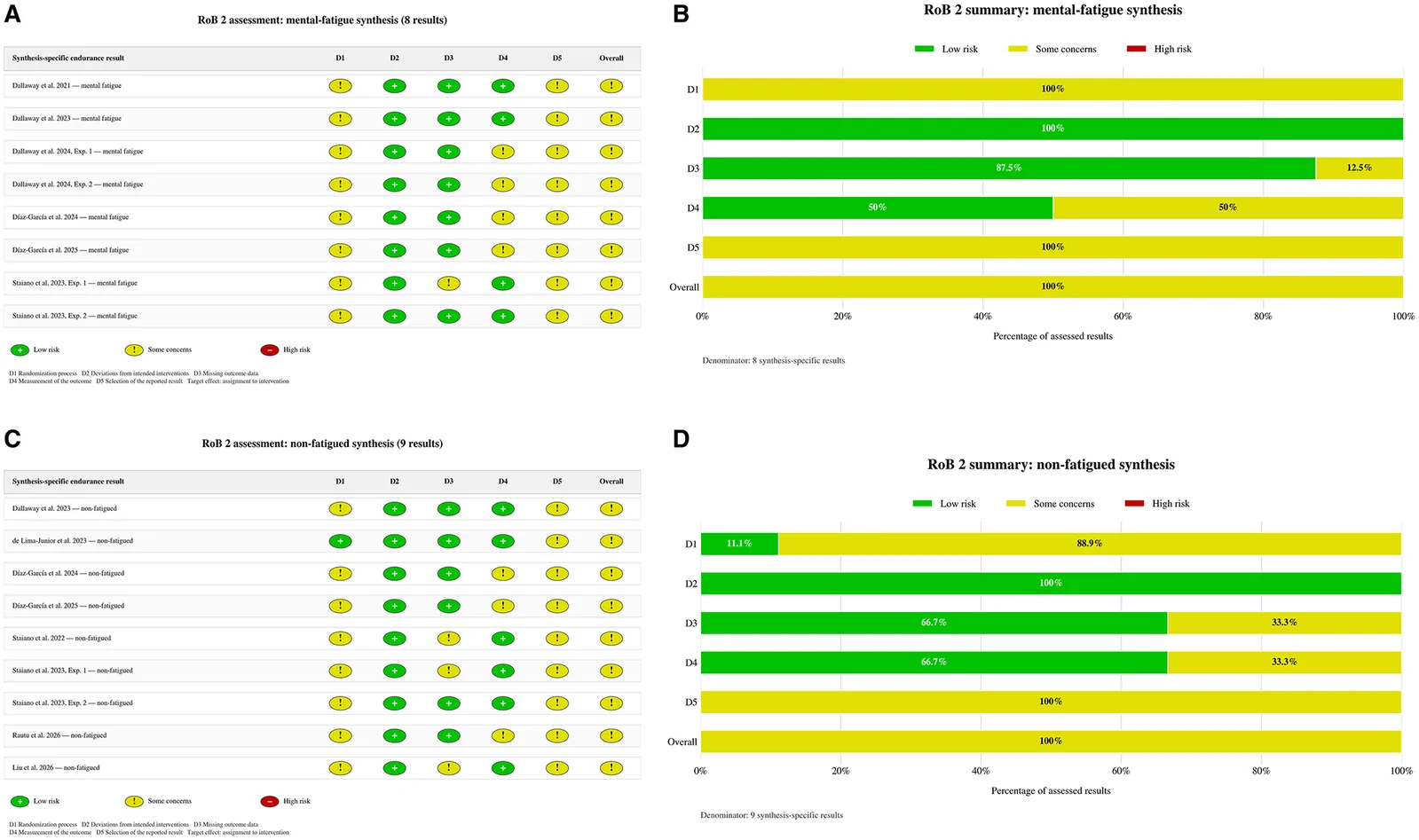
**(A)** Risk-of-bias judgments for endurance-performance results under mental-fatigue conditions. **(B)** Summary of risk-of-bias judgments for endurance-performance results under mental-fatigue conditions. **(C)** Risk-of-bias judgments for endurance-performance results under non-fatigued conditions. **(D)** Summary of risk-of-bias judgments for endurance-performance results under non-fatigued conditions.

Risk of bias was assessed using RoB 2 for each endurance-performance result included in the syntheses. Assessments were conducted separately for the eight mental-fatigue and nine non-fatigued estimates. All 17 results were judged as having some concerns overall, and none was judged to be at high risk of bias. Concerns primarily related to insufficient reporting of the randomization process, missing outcome data in some trials, lack of assessor blinding for outcomes requiring judgment, and the absence of sufficiently detailed prospective analysis plans. Figures 2A, B present the result-level risk-of-bias judgments and corresponding summary distribution for the mental-fatigue synthesis, respectively. Figures 2C, D present the corresponding assessments for the non-fatigued synthesis.

**Figure 3:**
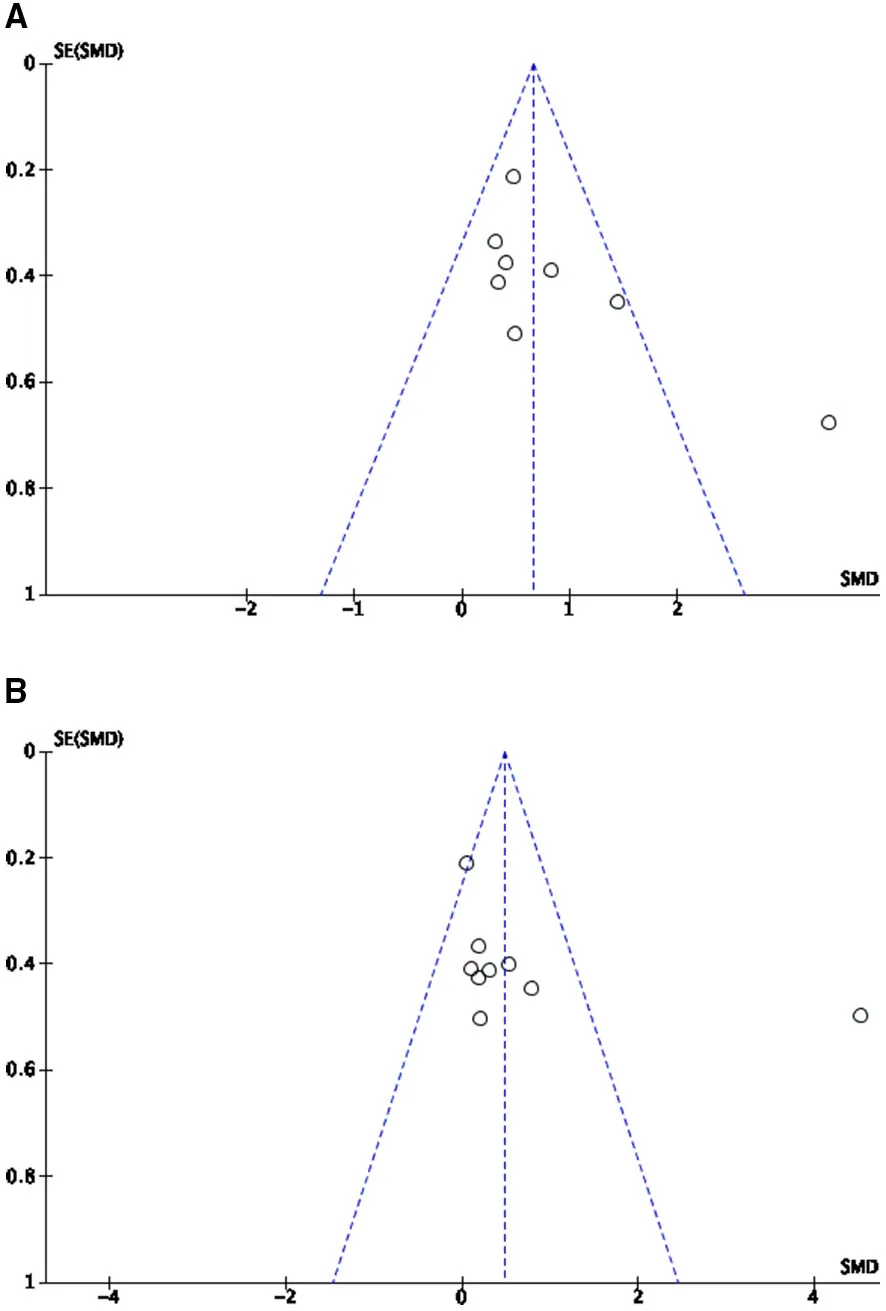
**(A)** Funnel plot for the mental-fatigue-condition synthesis the vertical dashed line indicates the fixed-effect estimate (SMD = 0.65; primary random-effects estimate = 0.82); diagonal dashed lines indicate pseudo 95% confidence limits. Descriptive only because *k* < 10. **(B)** Funnel plot for the non-fatigued-condition synthesis the vertical dashed line indicates the fixed-effect estimate (SMD = 0.48; primary random-effects estimate = 0.73); diagonal dashed lines indicate pseudo 95% confidence limits. Descriptive only because *k* < 10.

Funnel plots and Egger's regression were examined as exploratory assessments of small-study effects. Visual inspection did not show a clear and consistent pattern of asymmetry in either condition, although isolated large estimates produced some imbalance. Egger's regression did not indicate statistically significant small-study effects under mental-fatigue conditions (*k* = 8; *t* = 1.93, df = 6, *p* = 0.101; Figure 3A) or non-fatigued conditions (*k* = 9; *t* = 1.36, df = 7, *p* = 0.216; Figure 3B). Because both syntheses contained fewer than 10 independent experiments, these findings had low power and do not permit a reliable determination of the presence or absence of publication bias.

### 3.4 Main effects

The condition-specific meta-analyses included eight independent-experiment estimates under mental-fatigue conditions and nine under non-fatigued conditions. Some experiments contributed data to both condition-specific models, resulting in overlap between these counts. Effect selection and shared-sample handling are reported in Supplementary File S2.

#### 3.4.1 Endurance performance under conditions of mental fatigue

**Figure 4:**
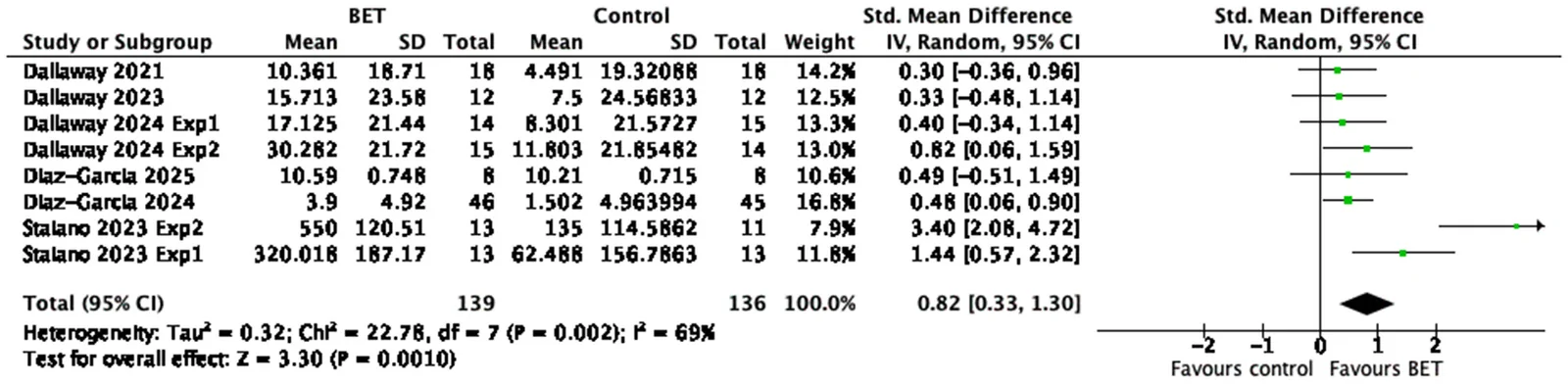
Forest plot of the effect of BET on physical endurance under conditions of mental fatigue.

Eight independent experiments (total analyzed *n* = 275) indicated a positive effect of BET on endurance performance under mental-fatigue conditions in the random-effects model (SMD = 0.82, 95% CI 0.33 to 1.30; *p* = 0.001; τ^2^ = 0.319; *Q*(7) = 22.78, *p* = 0.002; *I*^2^ = 69%; Figure 4). Heterogeneity was substantial.

#### 3.4.2 Endurance performance under non-fatigued condition

**Figure 5:**
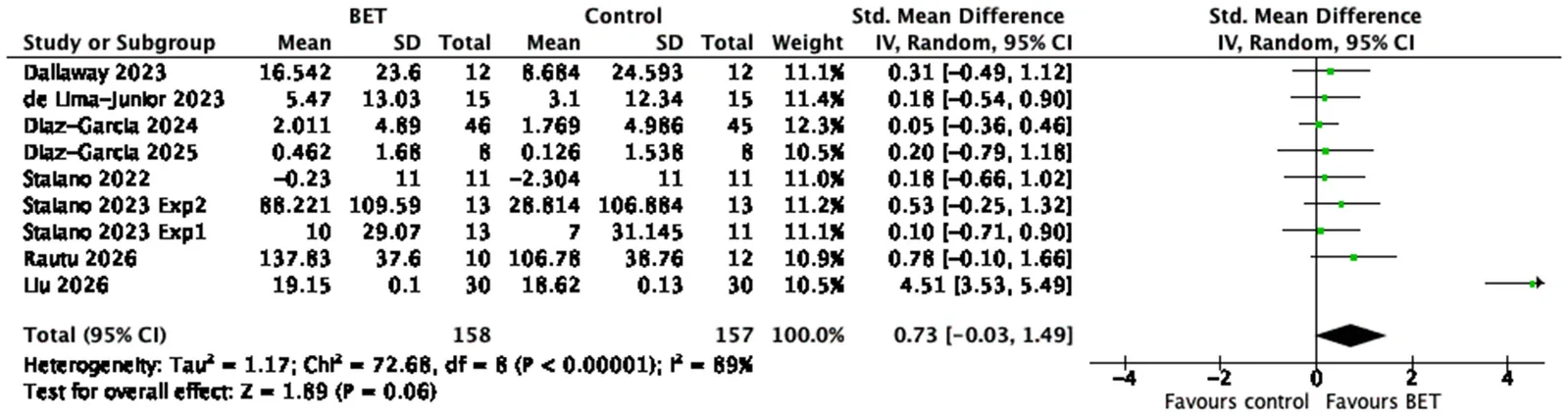
Effect of BET on physical endurance under non-fatigued conditions.

Nine independent experiments (total analyzed *n* = 315) yielded an imprecise, non-significant random-effects estimate under non-fatigued conditions (SMD = 0.73, 95% CI −0.03 to 1.49; *p* = 0.058; τ^2^ = 1.170; *Q*(8) = 72.68, *p* < 0.001; *I*^2^ = 89%; Figure 5). Heterogeneity was considerable, and influence analysis identified one extreme standardized effect as the principal source of the change.

### 3.5 Analysis of specificity of results by type of endurance

To explore differences across endurance domains, aerobic and muscular endurance were synthesized separately under mental-fatigue and non-fatigued conditions. Anaerobic endurance could not be pooled under mental-fatigue conditions because fewer than two independent experiments were available; under non-fatigued conditions, two independent experiments contributed to an exploratory pooled estimate. A multilevel meta-regression using non-fatigued conditions as the reference estimated a mental-fatigue vs. non-fatigued contrast of 0.59 (95% CI −0.30 to 1.48; *p* = 0.120). The model included 17 condition-specific effect estimates from 12 independent experiments and used experiment-clustered CR2 robust inference. The test did not provide statistically significant evidence that test state modified the BET effect; however, the estimate was imprecise because the number of clusters was small.

#### 3.5.1 Effect of BET on different endurance types under mental fatigue conditions

**Figure 6:**
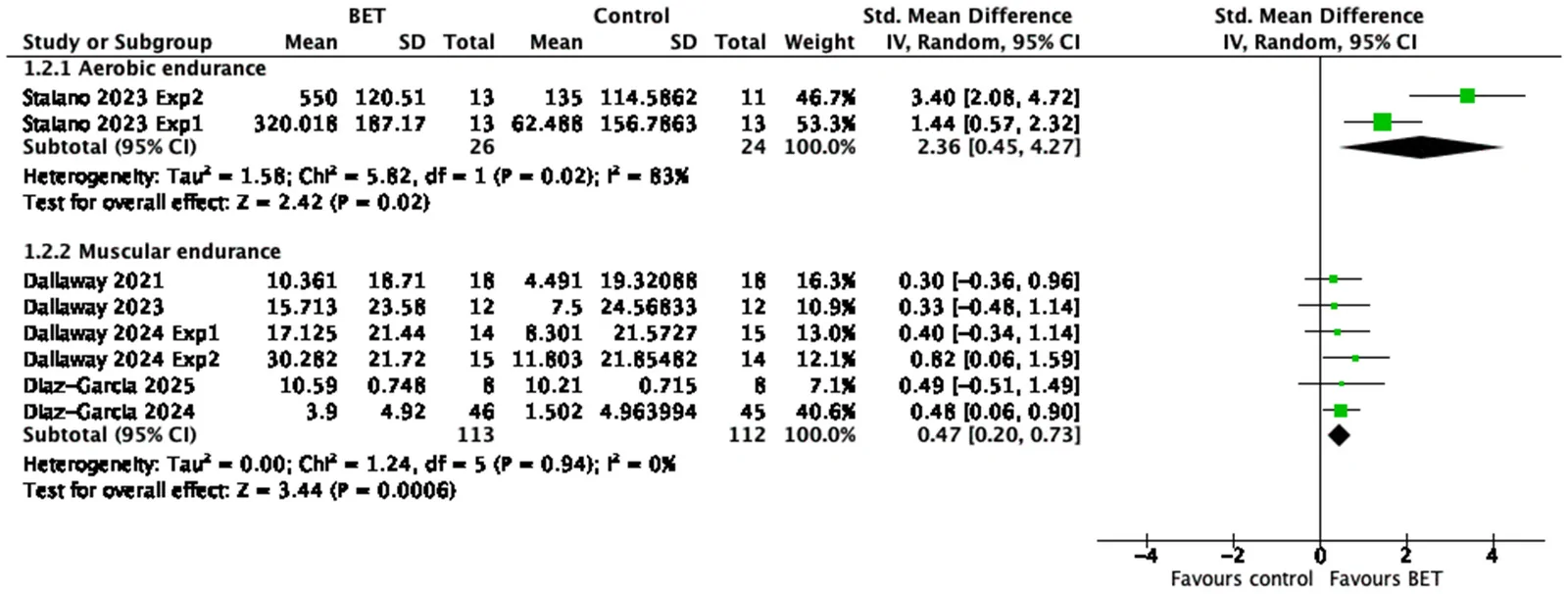
Forest plot of the effect of BET on different endurance types under conditions of mental fatigue.

Under mental-fatigue conditions, aerobic endurance was represented by two independent experiments (*n* = 50) and yielded a large but heterogeneous estimate (SMD = 2.36, 95% CI 0.45 to 4.27; *p* = 0.02; *I*^2^ = 83%) (Figure 6). Muscular endurance was represented by six independent experiments and yielded a smaller, consistent estimate (SMD = 0.47, 95% CI 0.20 to 0.73; *p* = 0.0006; *I*^2^ = 0%). Anaerobic endurance could not be synthesized under mental-fatigue conditions because fewer than two independent experiments were available.

#### 3.5.2 Effect of BET on different endurance types under non-fatigued conditions

**Figure 7:**
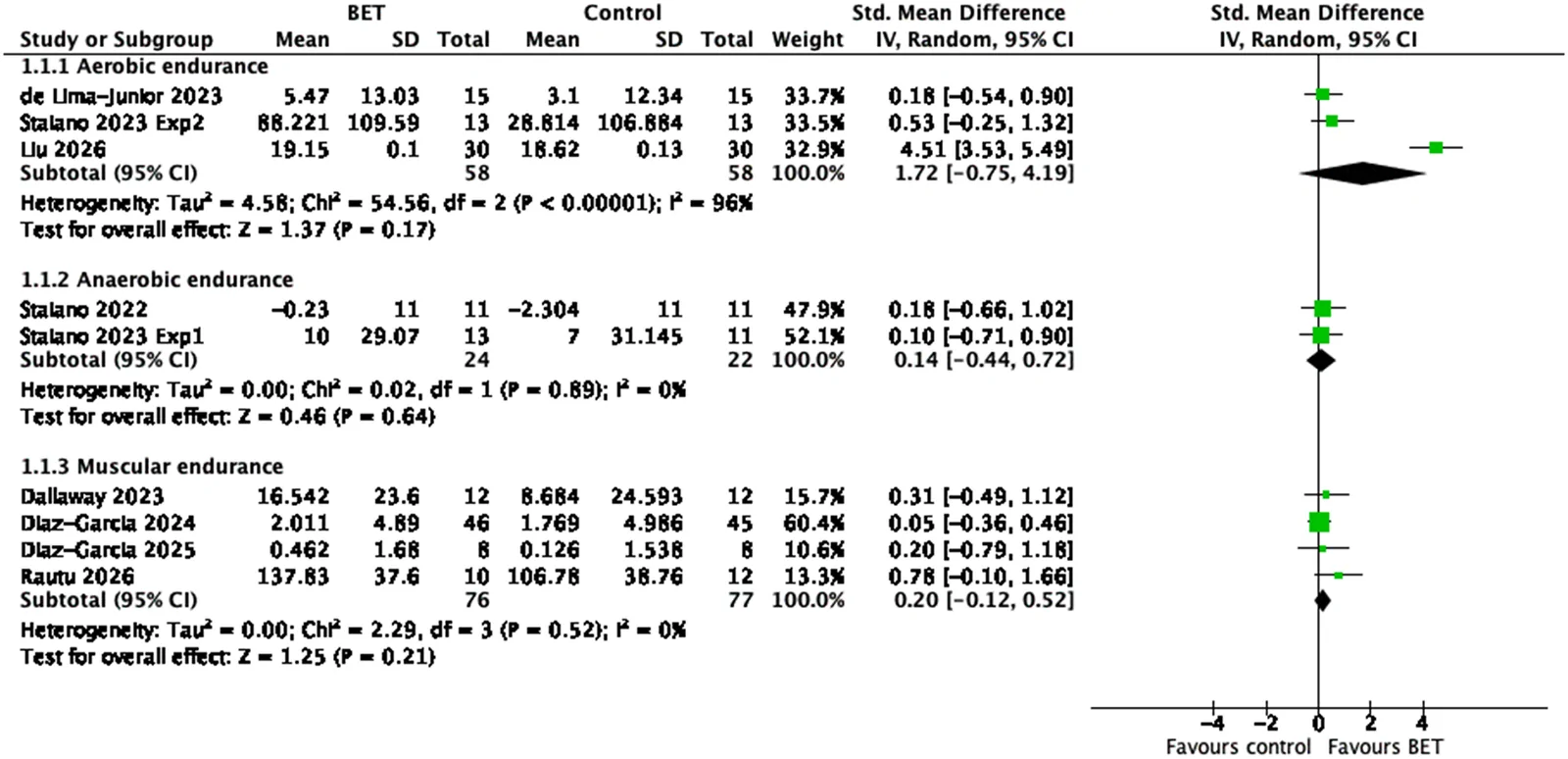
Forest plot of the effect of BET on different endurance types under non-fatigued conditions.

Under non-fatigued conditions, aerobic endurance was represented by three independent experiments (*n* = 116; SMD = 1.72, 95% CI −0.75 to 4.19; *p* = 0.172; *I*^2^ = 96%), while an exploratory pooled estimate from two independent experiments was available for anaerobic endurance (*n* = 46; SMD = 0.14, 95% CI −0.44 to 0.72; *p* = 0.64; *I*^2^ = 0%) (Figure 7). Muscular endurance was represented by four independent experiments (*n* = 153; SMD = 0.20, 95% CI −0.12 to 0.52; *p* = 0.21; *I*^2^ = 0%; Figure 7). The aerobic estimate was highly heterogeneous and strongly influenced by one extreme standardized effect, and the anaerobic estimate was too sparse for firm inference.

### 3.6 Certainty of evidence

GRADE certainty ranged from very low to moderate (Table 3). Certainty was very low for aerobic endurance under mental-fatigue conditions, moderate for muscular endurance under mental-fatigue conditions, very low for aerobic and anaerobic endurance under non-fatigued conditions, and low for muscular endurance under non-fatigued conditions. Downgrading decisions reflected RoB 2 concerns, inconsistency, imprecision, and the limited ability to assess publication bias with few experiments.

**Table 3:** Certainty of evidence.

Outcome	Analyzed n (no. of exp.)	Risk of bias	Inconsistency (*I*^2^)	Indirectness	Imprecision	Publication bias	Overall certainty/ Importance
Aerobic endurance under mental-fatigue conditions	50 (2 experiments)	Serious (−1) RoB 2: some concerns	Serious (−1) *I*^2^ = 83%	Not serious Direct PICOS evidence	Serious (−1) *n* = 50; wide CI	Not reliably assessable (< 10 independent experiments)	⊕°°° Very low critical
Muscular endurance under mental-fatigue conditions	225 (6 experiments)	Serious (−1) RoB 2: some concerns	Not serious *I*^2^ = 0%	Not serious Direct PICOS evidence	Not serious CI excludes no effect	Not reliably assessable (6 independent experiments)	⊕⊕⊕° Moderate critical
Aerobic endurance under non-fatigued conditions	116 (3 experiments)	Serious (−1) RoB 2: some concerns	Very serious (−2) *I*^2^ = 96%	Not serious Direct PICOS evidence	Serious (−1) Small evidence base; wide CI	Not reliably assessable (< 10 independent experiments; one extreme effect)	⊕°°° Very low critical
Anaerobic endurance under non-fatigued conditions	46 (2 experiments)	Serious (−1) RoB 2: some concerns	Not serious *I*^2^ = 0%	Not serious Direct PICOS evidence	Very serious (−2) *n* = 46; CI crosses no effect and appreciable benefit/harm	Not reliably assessable (< 10 independent experiments)	⊕°°° Very low critical
Muscular endurance under non-fatigued conditions	153 (4 experiments)	Serious (−1) RoB 2: some concerns	Not serious *I*^2^ = 0%	Not serious Direct PICOS evidence	Serious (−1) *n* = 153; CI crosses no effect	Not reliably assessable (4 independent experiments)	⊕⊕°° Low critical
Effect estimates and reasons for downgrading or not downgrading							
Outcome		Effect estimate (95% CI)		GRADE rationale			
Aerobic endurance under mental-fatigue conditions		SMD 2.36 higher (95% CI 0.45 to 4.27 higher)		Downgraded one level each for risk of bias, inconsistency, and imprecision. No downgrade for indirectness. Publication bias could not be assessed reliably with two experiments.			
Muscular endurance under mental-fatigue conditions		SMD 0.47 higher (95% CI 0.20 to 0.73 higher)		Downgraded one level for risk of bias. No downgrade for inconsistency, indirectness, or imprecision; the confidence interval excluded no effect. Publication-bias assessment remained limited.			
Aerobic endurance under non-fatigued conditions		SMD 1.72 higher (95% CI 0.75 lower to 4.19 higher)		Downgraded for risk of bias, very serious inconsistency, and imprecision. No additional publication-bias downgrade was applied because the exploratory assessment involved fewer than 10 experiments, although one extreme effect was present.			
Anaerobic endurance under non-fatigued conditions		SMD 0.14 higher (95% CI 0.44 lower to 0.72 higher)		Downgraded one level for risk of bias and two levels for very serious imprecision. No downgrade for inconsistency or indirectness; publication bias was not assessable.			
Muscular endurance under non-fatigued conditions		SMD 0.20 higher (95% CI 0.12 lower to 0.52 higher)		Downgraded one level for risk of bias and one level for imprecision because the confidence interval crossed no effect. No downgrade for inconsistency or indirectness.			

RCTs started at high certainty. Serious limitations downgraded certainty by one level and very serious limitations by two levels. Positive SMD values favor BET. Funnel-plot asymmetry and Egger testing were not considered reliable for outcome groups with fewer than 10 independent experiments.

### 3.7 Sensitivity analysis

For endurance performance under mental-fatigue conditions (8 independent experiments, *n* = 275), the random-effects model yielded an SMD of 0.82 (95% CI 0.33 to 1.30; *p* = 0.001; *I*^2^ = 69%). Leave-one-out estimates ranged from 0.55 to 0.91 and remained statistically significant after every exclusion; *I*^2^ ranged from 0% to 74%. Omitting Staiano et al. (2023 Exp1), which had the largest individual estimate, reduced the pooled effect to SMD = 0.55 (95% CI 0.29 to 0.80; *p* < 0.001) and *I*^2^ to 0%. The fixed-effect estimate was SMD = 0.65 (95% CI 0.40 to 0.90; *p* < 0.001). Thus, the direction and statistical significance were stable, but the magnitude and heterogeneity were influenced by one experiment.

For endurance performance under non-fatigued conditions (9 independent experiments, *n* = 315), the random-effects model yielded SMD = 0.73 (95% CI −0.03 to 1.49; *p* = 0.058; *I*^2^ = 89%). Leave-one-out estimates ranged from 0.22 to 0.83 and remained non-significant at the 0.05 level. Omitting Liu et al. (2026), whose large standardized estimate reflected a large mean difference relative to the reported pooled SD, reduced the pooled estimate to SMD = 0.22 (95% CI −0.03 to 0.47; *p* = 0.080) and *I*^2^ to 0%. The fixed-effect estimate was SMD = 0.48 (95% CI 0.24 to 0.72; *p* < 0.001). The non-fatigued result was sensitive to one estimate and to model choice, leaving the effect uncertain.

## 4 Discussion

### 4.1 Principal findings and relation to previous evidence

The random-effects meta-analysis suggests that BET may improve endurance performance when outcomes are assessed after mental-fatigue induction (8 independent experiments, *n* = 275; SMD = 0.82, 95% CI 0.33 to 1.30; *I*^2^ = 69%). Under non-fatigued conditions, the pooled estimate was imprecise and highly heterogeneous (9 independent experiments, *n* = 315; SMD = 0.73, 95% CI −0.03 to 1.49; *p* = 0.058; *I*^2^ = 89%). A formal multilevel moderator analysis did not identify a statistically significant difference between test states (contrast = 0.59, 95% CI −0.30 to 1.48; *p* = 0.120). The available data therefore do not establish that BET has different effects under mental-fatigue and non-fatigued conditions.

The mental-fatigue estimate is similar to that reported by Yu et al. (2026) (SMD = 0.87, 95% CI 0.61 to 1.13), whereas their non-fatigued estimate was smaller (SMD = 0.21, 95% CI 0.08 to 0.34). Differences in eligibility criteria, endpoint selection, treatment of multi-experiment articles, and statistical models may explain the discrepancy. Qing et al. (2026) likewise reported potential benefits for physical and cognitive performance but emphasized small samples and substantial protocol heterogeneity. The present endurance-specific analysis is consistent with a possible benefit after mental-fatigue induction, while showing that evidence for a between-state difference remains insufficient.

### 4.2 Interpretation of the condition-specific findings

The mental-fatigue estimate was positive, but its magnitude was uncertain. Leave-one-out estimates remained positive (SMD range 0.55 to 0.91), although removal of one influential experiment reduced *I*^2^ from 69% to 0% and the pooled effect to SMD = 0.55 (95% CI 0.29 to 0.80). Aerobic endurance was represented by only two experiments and yielded a large, heterogeneous, very-low-certainty estimate (SMD = 2.36; *I*^2^ = 83%). Muscular endurance was represented by six experiments (*n* = 225) and yielded a smaller, more consistent estimate (SMD = 0.47; *I*^2^ = 0%) with moderate certainty. Comparative effects across endurance domains remain uncertain.

Evidence under non-fatigued conditions remains inconclusive. The aerobic estimate was imprecise and highly heterogeneous (3 independent experiments, *n* = 116; SMD = 1.72, 95% CI −0.75 to 4.19; *I*^2^ = 96%), while the exploratory anaerobic estimate was small and imprecise (2 independent experiments, *n* = 46; SMD = 0.14, 95% CI −0.44 to 0.72; *I*^2^ = 0%) and the muscular estimate was also small (4 independent experiments, *n* = 153; SMD = 0.20, 95% CI −0.12 to 0.52; *I*^2^ = 0%). One extreme standardized value strongly influenced the overall pooled estimate: its removal reduced the effect from SMD = 0.73 to 0.22 and *I*^2^ from 89% to 0%. The difference between fixed-effect and random-effects estimates further indicates that the non-fatigued result is not sufficiently stable for a definitive conclusion.

### 4.3 Possible neurobiological and psychophysiological mechanisms

Mental fatigue can increase perceived effort, reduce willingness to sustain effort, and impair executive control (Marcora et al., 2009; Boksem and Tops, 2008). Repeated exposure to combined cognitive and physical demands may improve tolerance of cognitive effort or help maintain task-relevant control during subsequent endurance performance (André et al., 2025). Prefrontal and anterior cingulate networks involved in inhibitory control, effort valuation, and allocation of cognitive resources provide plausible neural targets. Differences in prefrontal oxygenation, hemodynamic responses, or structure-function coupling reported in selected BET and dual-task studies may reflect altered cortical recruitment (Dallaway et al., 2023; Liu et al., 2026; Kimura et al., 2022; De Wachter et al., 2021). However, these findings come from small, heterogeneous studies, and the physiological measures are sensitive to systemic influences; direct mediation of endurance improvement has not been demonstrated.

Autonomic regulation may also contribute, although evidence linking changes in heart-rate variability to BET-related performance gains is limited (Forte et al., 2019). Inconsistent changes in maximal oxygen uptake, blood lactate, and heart rate suggest that the observed performance effects are not readily explained by a single peripheral physiological adaptation. Studies that repeatedly measure perceived effort, motivation, executive control, autonomic function, and cerebral hemodynamics could clarify the temporal and mediating pathways involved.

### 4.4 Strengths and limitations of the study

The experiments varied in participant characteristics, cognitive tasks, physical-training modes, fatigue manipulations, intervention duration, and endurance endpoints. One estimate per independent experiment was retained within each condition; dependence between repeated test states was addressed in the moderator analysis using experiment-clustered robust inference. Nevertheless, covariance information was generally unavailable, limiting more detailed modeling of correlated outcomes. Funnel plots and Egger tests were exploratory because each model contained fewer than 10 experiments and therefore had limited power.

This review is limited by the small number and size of independent experiments, concentration of evidence within a few research groups, risk-of-bias concerns, and certainty ratings ranging from very low to moderate. Clinical and methodological heterogeneity reduces the interpretability of pooled SMDs. Covariance among repeated conditions and multiple outcomes was generally unavailable; dependence across conditions was therefore addressed using experiment-clustered CR2 robust inference, but only 12 experiment clusters were available and the moderator estimate remained imprecise. The non-fatigued result was sensitive to one extreme effect and model choice, subgroup analyses were underpowered, mechanistic outcomes were not synthesized quantitatively, and publication-bias assessments had low power. Restriction to published English-language reports and limited representation of women, clinical populations, and elite athletes further constrain generalizability.

### 4.5 Future research

Future trials should be prospectively registered, adequately powered, and preferably multicenter. Primary outcomes and endpoint-selection rules should be specified in advance, and reports should provide analyzed sample sizes, means, SDs, change-score correlations, attrition, and covariance among repeated fatigue-state outcomes. Standardized fatigue induction and a prespecified group-by-time-by-state interaction are needed to test effect modification. Mechanistic studies should combine endurance outcomes with validated measures of perceived effort, motivation, cognitive control, autonomic regulation, and cerebral hemodynamics, while randomized head-to-head comparisons are required to determine whether intervention dose or protocol influences efficacy.

## 5 Conclusion

BET may improve endurance performance after mental-fatigue induction, but the magnitude remains uncertain because the evidence is heterogeneous and influenced by a small number of experiments. Evidence under non-fatigued conditions is inconclusive, and the formal moderator test did not establish a difference between test states. Neurobiological and psychophysiological mechanisms require direct evidence. At present, BET may be considered an exploratory adjunct to physical training, with confirmation needed from adequately powered trials incorporating interaction and mechanistic analyses.

## Data Availability

The original contributions presented in the study are included in the article/Supplementary material, further inquiries can be directed to the corresponding author/s.
