# Supplementary material for: The effects of brain endurance training on physical endurance performance under mental fatigue and non-fatigued conditions: a systematic review and meta-analysis
Source: Front Psychol. 2026 Sep 11;17:1858200. doi: 10.3389/fpsyg.2026.1858200 (PMC13612342; doi:10.3389/fpsyg.2026.1858200)
Supplement: Supplementary file 1 [file Table_1.docx]

# Search framework

**Search execution:** PubMed; Web of Science, Scopus, and Cochrane Library

**Language:** English where a database language limit was supported; otherwise assessed during screening.

**Design handling:** No RCT filter was applied in PubMed, Web of Science, or Scopus to reduce the risk of missing eligible trials. RCT status and PICOS eligibility were determined during screening.

**Strategy development:** Terms were derived from known BET reports and expanded to cover brain endurance, combined/concurrent cognitive-physical, dual-task, cognitive-motor, and multimodal training, paired with specific endurance and exercise-performance outcomes.

**Fatigue concept:** Mental fatigue was not required because eligible trials also assessed endurance under non-fatigue conditions; fatigue status was classified during screening and data extraction.

**Broad performance term:** The unrestricted word ‘performance’ was not searched alone; only the specific phrases ‘physical performance’ and ‘exercise performance’ were used.

**Deduplication:** All 776 exports were stored in Zotero Desktop 9.0.4. Exact DOI, PMID, normalized title plus year, and normalized title matching yielded 358 records; manual review of two high-similarity pairs identified one additional duplicate, leaving 357 unique records.

# Database overview

| **Database** | **Primary fields** | **Date handling** | **Design handling** |
| --- | --- | --- | --- |
| **PubMed** | Title/Abstract | Explicit date range | Screening stage |
| **Web of Science** | Topic | Explicit DOP range | Screening stage |
| **Scopus** | Title/Abstract/Keywords | Year + manual cut-off | Screening stage |
| **Cochrane Library** | Title/Abstract/Keyword | Manual cut-off | Trials content type |

# 1. PubMed

**Interface:** PubMed

**Search field(s):** Title/Abstract [tiab]; Publication Date [dp]; Language [la]

**Limits:** English; database inception through 5 August 2026; no RCT, full-text, or population filter.

**Controlled vocabulary:** No BET-specific MeSH term was available; explicit Title/Abstract text words were used.

**Truncation / variants:** None. Hyphenated, non-hyphenated, and phrase variants were enumerated explicitly.

**Actual search date:** 5 August 2026

**Records retrieved / exported:** 165 / 165

## Full search strategy

(("brain endurance training"[tiab] OR "brain endurance"[tiab] OR "cognitive physical training"[tiab] OR "cognitive-physical training"[tiab] OR "combined cognitive and physical training"[tiab] OR "combined cognitive physical training"[tiab] OR "concurrent cognitive and physical training"[tiab] OR "simultaneous cognitive and physical training"[tiab] OR "dual-task training"[tiab] OR "dual task training"[tiab] OR "cognitive-motor training"[tiab] OR "cognitive motor training"[tiab] OR "multimodal training"[tiab] OR "multi-modal training"[tiab]) AND (endurance[tiab] OR "physical performance"[tiab] OR "exercise performance"[tiab] OR aerobic[tiab] OR anaerobic[tiab] OR "muscular endurance"[tiab] OR "time to exhaustion"[tiab] OR "time-to-exhaustion"[tiab] OR "exercise tolerance"[tiab])) AND ("1800/01/01"[dp] : "2026/08/05"[dp]) AND english[la]

**Implementation note.** Each synonym is explicitly tagged because field tags suppress PubMed Automatic Term Mapping.

# 2. Web of Science Core Collection

**Interface:** Web of Science Advanced Search

**Search field(s):** Topic TS (title, abstract, author keywords, Keywords Plus); DOP; LA

**Limits:** English; DOP through 5 August 2026; no RCT or document-type filter.

**Controlled vocabulary:** Not applicable; Topic searching uses title, abstract, author keywords, and Keywords Plus.

**Truncation / variants:** None. Hyphenated, non-hyphenated, and phrase variants were enumerated explicitly.

**Actual search date:** 6 August 2026

**Records retrieved / exported:** 218 / 218

## Full search strategy

TS=(("brain endurance training" OR "brain endurance" OR "cognitive physical training" OR "cognitive-physical training" OR "combined cognitive and physical training" OR "combined cognitive physical training" OR "concurrent cognitive and physical training" OR "simultaneous cognitive and physical training" OR "dual-task training" OR "dual task training" OR "cognitive-motor training" OR "cognitive motor training" OR "multimodal training" OR "multi-modal training") AND (endurance OR "physical performance" OR "exercise performance" OR aerobic OR anaerobic OR "muscular endurance" OR "time to exhaustion" OR "time-to-exhaustion" OR "exercise tolerance")) AND DOP=1800-01-01/2026-08-05 AND LA=(English)

**Implementation note.** Run in the Web of Science Core Collection. The TS field searches titles, abstracts, author keywords, and Keywords Plus.

# 3. Scopus

**Interface:** Scopus Advanced Search

**Search field(s):** TITLE-ABS-KEY (title, abstract, keywords); PUBYEAR; LANGUAGE

**Limits:** English; PUBYEAR < 2027; records published after 5 August 2026 were excluded manually; no RCT filter.

**Controlled vocabulary:** Not applicable; Scopus does not use a controlled thesaurus for TITLE-ABS-KEY searching.

**Truncation / variants:** None. Hyphenated, non-hyphenated, and phrase variants were enumerated explicitly.

**Actual search date:** 6 August 2026

**Records retrieved / exported:** 217 / 217

## Full search strategy

TITLE-ABS-KEY(("brain endurance training" OR "brain endurance" OR "cognitive physical training" OR "cognitive-physical training" OR "combined cognitive and physical training" OR "combined cognitive physical training" OR "concurrent cognitive and physical training" OR "simultaneous cognitive and physical training" OR "dual-task training" OR "dual task training" OR "cognitive-motor training" OR "cognitive motor training" OR "multimodal training" OR "multi-modal training") AND (endurance OR "physical performance" OR "exercise performance" OR aerobic OR anaerobic OR "muscular endurance" OR "time to exhaustion" OR "time-to-exhaustion" OR "exercise tolerance")) AND PUBYEAR < 2027 AND LANGUAGE(english)

**Implementation note.** Because the strategy limits by publication year, records from 2026 must be screened manually against the planned cut-off date.

# 4. Cochrane Library

**Interface:** Cochrane Library Search Manager

**Search field(s):** Title, Abstract, Keyword (:ti,ab,kw)

**Limits:** Content type: Trials; all dates; records published after 5 August 2026 were excluded manually; language, design, and PICOS were assessed during screening.

**Controlled vocabulary:** No BET-specific subject heading was applied; explicit Title/Abstract/Keyword text words were used.

**Truncation / variants:** None. Hyphenated, non-hyphenated, and phrase variants were enumerated explicitly.

**Actual search date:** 6 August 2026

**Records retrieved / exported:** 176 / 176

## Full search strategy

#1 ("brain endurance training" OR "brain endurance" OR "cognitive physical training" OR "cognitive-physical training" OR "combined cognitive and physical training" OR "combined cognitive physical training" OR "concurrent cognitive and physical training" OR "simultaneous cognitive and physical training" OR "dual-task training" OR "dual task training" OR "cognitive-motor training" OR "cognitive motor training" OR "multimodal training" OR "multi-modal training"):ti,ab,kw

#2 (endurance OR "physical performance" OR "exercise performance" OR aerobic OR anaerobic OR "muscular endurance" OR "time to exhaustion" OR "time-to-exhaustion" OR "exercise tolerance"):ti,ab,kw

#3 #1 AND #2

**Implementation note.** Enter the strategy as three separate lines in Search Manager, then limit the content type to Trials.
