# Supplementary material for: The effects of brain endurance training on physical endurance performance under mental fatigue and non-fatigued conditions: a systematic review and meta-analysis
Source: Front Psychol. 2026 Sep 11;17:1858200. doi: 10.3389/fpsyg.2026.1858200 (PMC13612342; doi:10.3389/fpsyg.2026.1858200)
Supplement: Supplementary file 2 [file Table_2.docx]

# S2.1 Purpose

This supplement documents the effect-size dataset used for the updated overall analyses, the rules used to select and process effect estimates, and complete leave-one-out influence analyses. Its purpose is to assess whether any single independent experiment materially changes the pooled standardized mean difference (SMD) or heterogeneity.

# S2.2 Unit of analysis and effect-size handling

| **Item** | **Prespecified handling rule** |
| --- | --- |
| Unit of analysis | The primary unit was an independent experiment within a prespecified analysis condition, not the article. Multi-experiment articles were separated by Experiment_ID. |
| Conditions | Mental-fatigue and non-fatigued conditions were synthesized separately. Effects from the same participants across the two conditions were not combined in one overall model. |
| Outcome selection | The primary overall model retained one endurance-performance effect per independent experiment within each condition. Multiple candidate outcomes from a shared sample were not treated as independent effects in the same overall model. Domain-specific alternatives should be reported in subgroup or additional sensitivity analyses. |
| Effect metric | Continuous outcomes measured on different scales were expressed as standardized mean differences with small-sample correction (Hedges’ g), using the analyzed BET and control sample sizes and the corresponding means and SDs. Extracted SMD and SE values were retained when they were the verified analysis inputs. |
| Direction | All effects were oriented so that a positive SMD favors BET. Outcomes for which a lower numerical value indicates better performance were sign-reversed before pooling. |
| Dispersion data | SDs were used as reported. Values converted from other dispersion statistics or digitized from figures were identified in the extraction sheet with the conversion formula and source location. Source-level verification confirmed that the Liu et al. (2026) dispersion values were SDs rather than SEs; no correction to the extracted data was required. |
| Shared samples | Where several outcomes share the same participants, the shared-sample flag must be retained. Such effects must not be entered as independent observations in a conventional two-level overall meta-analysis. |
| Model | The primary analysis used a DerSimonian–Laird random-effects model. A fixed-effect model was calculated only as a model-sensitivity check. Two-sided p < 0.05 was used for statistical significance. |
| Influence analysis | Each independent experiment was removed once. Changes in pooled SMD, 95% CI, p value, τ², and I² were examined. A marked fall in I² or pooled SMD was treated as evidence of influence, not as a reason for automatic exclusion. |

Source-level verification was completed for Liu et al. (2026). The extracted endpoint was post-intervention maximum speed during the 30–15 Intermittent Fitness Test, reported in km·h⁻¹. Dispersion values were confirmed as SDs rather than SEs: BET, 19.15 ± 0.10 (n = 30); control, 18.62 ± 0.13 (n = 30). No correction to the extracted data was required.

# S2.3 Effect estimates entered in the influence analysis

Table S2.1 lists the effect estimates used in the two overall models. Positive values favor BET. Values are shown to three decimals; calculations used unrounded values.

| **Condition** | **Independent experiment** | **SMD** | **SE** | **Influence/data note** |
| --- | --- | --- | --- | --- |
| Mental fatigue | Dallaway 2021 | 0.302 | 0.335 |  |
| Mental fatigue | Dallaway 2023 | 0.329 | 0.412 |  |
| Mental fatigue | Dallaway 2024 Exp1 | 0.399 | 0.376 |  |
| Mental fatigue | Dallaway 2024 Exp2 | 0.824 | 0.389 |  |
| Mental fatigue | Díaz-García 2024 | 0.481 | 0.213 |  |
| Mental fatigue | Díaz-García 2025 | 0.491 | 0.510 |  |
| Mental fatigue | Staiano 2023 Exp2 | 3.400 | 0.675 | Influential: largest SMD; drives heterogeneity |
| Mental fatigue | Staiano 2023 Exp1 | 1.445 | 0.448 |  |
| Non-fatigued | Dallaway 2023 | 0.315 | 0.411 |  |
| Non-fatigued | de Lima-Junior 2023 | 0.182 | 0.366 |  |
| Non-fatigued | Díaz-García 2024 | 0.049 | 0.210 |  |
| Non-fatigued | Díaz-García 2025 | 0.197 | 0.502 |  |
| Non-fatigued | Staiano 2022 | 0.181 | 0.427 |  |
| Non-fatigued | Staiano 2023 Exp1 | 0.531 | 0.400 |  |
| Non-fatigued | Staiano 2023 Exp2 | 0.096 | 0.410 |  |
| Non-fatigued | Rautu 2026 | 0.781 | 0.447 |  |
| Non-fatigued | Liu 2026 | 4.511 | 0.498 | Influential: mean difference 0.53; pooled SD 0.116 |

# S2.4 Complete leave-one-out results

Table S2.2 reports the overall random-effects and fixed-effect models followed by every leave-one-out random-effects model. I² and τ² are recalculated after each exclusion.

| **Condition** | **Analysis** | **Omitted experiment** | **k** | **SMD** | **95% CI** | **p** | **I²** | **τ²** |
| --- | --- | --- | --- | --- | --- | --- | --- | --- |
| Mental-fatigue | Random effects | None (overall model) | 8 | 0.82 | 0.33 to 1.30 | <0.001 | 69.3% | 0.319 |
| Mental-fatigue | Fixed effect | None (overall model) | 8 | 0.65 | 0.40 to 0.90 | <0.001 | 69.3% | 0.000 |
| Mental-fatigue | Leave-one-out random effects | Dallaway 2021 | 7 | 0.91 | 0.36 to 1.47 | 0.001 | 72.1% | 0.387 |
| Mental-fatigue | Leave-one-out random effects | Dallaway 2023 | 7 | 0.90 | 0.35 to 1.45 | 0.001 | 72.9% | 0.375 |
| Mental-fatigue | Leave-one-out random effects | Dallaway 2024 Exp1 | 7 | 0.89 | 0.34 to 1.45 | 0.002 | 73.1% | 0.389 |
| Mental-fatigue | Leave-one-out random effects | Dallaway 2024 Exp2 | 7 | 0.83 | 0.27 to 1.39 | 0.004 | 73.4% | 0.391 |
| Mental-fatigue | Leave-one-out random effects | Diaz-Garcia 2024 | 7 | 0.91 | 0.30 to 1.52 | 0.003 | 72.5% | 0.477 |
| Mental-fatigue | Leave-one-out random effects | Diaz-Garcia 2025 | 7 | 0.87 | 0.33 to 1.41 | 0.002 | 73.5% | 0.369 |
| Mental-fatigue | Leave-one-out random effects | Staiano 2023 Exp2 | 7 | 0.55 | 0.29 to 0.80 | <0.001 | 0.0% | 0.000 |
| Mental-fatigue | Leave-one-out random effects | Staiano 2023 Exp1 | 7 | 0.73 | 0.22 to 1.24 | 0.005 | 69.0% | 0.304 |
| Non-fatigued | Random effects | None (overall model) | 9 | 0.73 | -0.03 to 1.49 | 0.058 | 89.0% | 1.170 |
| Non-fatigued | Fixed effect | None (overall model) | 9 | 0.48 | 0.24 to 0.72 | <0.001 | 89.0% | 0.000 |
| Non-fatigued | Leave-one-out random effects | Dallaway 2023 | 8 | 0.79 | -0.07 to 1.64 | 0.070 | 90.3% | 1.342 |
| Non-fatigued | Leave-one-out random effects | de Lima-Junior 2023 | 8 | 0.81 | -0.06 to 1.67 | 0.067 | 90.3% | 1.373 |
| Non-fatigued | Leave-one-out random effects | Diaz-Garcia 2024 | 8 | 0.83 | -0.08 to 1.74 | 0.074 | 89.4% | 1.546 |
| Non-fatigued | Leave-one-out random effects | Diaz-Garcia 2025 | 8 | 0.80 | -0.04 to 1.63 | 0.061 | 90.3% | 1.286 |
| Non-fatigued | Leave-one-out random effects | Staiano 2022 | 8 | 0.80 | -0.04 to 1.65 | 0.063 | 90.3% | 1.323 |
| Non-fatigued | Leave-one-out random effects | Staiano 2023 Exp1 | 8 | 0.76 | -0.10 to 1.62 | 0.082 | 90.4% | 1.354 |
| Non-fatigued | Leave-one-out random effects | Staiano 2023 Exp2 | 8 | 0.81 | -0.03 to 1.66 | 0.060 | 90.2% | 1.327 |
| Non-fatigued | Leave-one-out random effects | Rautu 2026 | 8 | 0.73 | -0.11 to 1.57 | 0.090 | 90.3% | 1.311 |
| Non-fatigued | Leave-one-out random effects | Liu 2026 | 8 | 0.22 | -0.03 to 0.47 | 0.080 | 0.0% | 0.000 |

# S2.5 Investigation of anomalous changes

| **Condition** | **Source** | **Why flagged** | **Observed influence** | **Verification/status** |
| --- | --- | --- | --- | --- |
| Mental fatigue | Staiano 2023 Exp2 | Individual SMD = 3.40, the largest effect in this condition. | Removing it changed SMD 0.82→0.55 and I² 69%→0%. | Retained in the primary analysis and identified as influential; the experiment label was corrected to Experiment 2. |
| Non-fatigued | Liu 2026 | BET 19.15 (SD 0.10) vs control 18.62 (SD 0.13); mean difference 0.53 relative to pooled SD 0.116 produced SMD = 4.51. | Removing it changed SMD 0.73→0.22 and I² 89%→0%. | Source-level verification confirmed that 0.10 and 0.13 were SDs, the endpoint was post-intervention maximum speed, and the unit was km·h⁻¹. No data correction was required. |
| Non-fatigued | Model comparison | The extreme value creates substantial between-experiment dispersion. | Random effects: 0.73 (−0.03 to 1.49); fixed effect: 0.48 (0.24 to 0.72). | The random-effects model was retained as the primary model, and the discrepancy between the random-effects and fixed-effect estimates was reported. |
| Mental fatigue | Forest-plot arithmetic check | The displayed study-level SMDs, including Staiano 2023 Exp2 = 3.40, were independently re-entered. | Final recalculation gave I² = 69% and a pooled random-effects SMD of 0.82 (95% CI 0.33 to 1.30). | The forest plot was regenerated from the verified dataset using the prespecified random-effects model, and the manuscript was updated to match. |
| Non-fatigued | Forest-plot arithmetic check | The displayed study-level SMDs, including Liu 2026 = 4.51, were independently re-entered. | Final recalculation gave I² = 89% and a pooled random-effects SMD of 0.73 (95% CI −0.03 to 1.49). | The forest plot was regenerated from the verified dataset using the prespecified random-effects model, and the manuscript was updated to match. |

# S2.6 Robustness conclusion

Mental-fatigue analysis: the positive direction and statistical significance persisted after every single-experiment exclusion, but the magnitude and heterogeneity were influenced by Staiano 2023 Exp2. The conclusion is robust for direction, not for exact magnitude or homogeneity.

Non-fatigued analysis: the random-effects result remained non-significant after every single-experiment exclusion, but the effect magnitude and heterogeneity were dominated by Liu et al. (2026), and the fixed-effect and random-effects models gave different significance decisions. The analysis is not robust with respect to effect magnitude, heterogeneity, or model choice. Source-level verification confirmed the endpoint, units, and that the dispersion values were SDs rather than SEs; no correction to the extracted data was required. The final forest plot was generated from the verified dataset.
